# Satisfaction and Workload as Predictors of Psychological Distress in Professionals of Psychosocial Care Centers During the COVID-19 Pandemic

**DOI:** 10.3390/nursrep14040290

**Published:** 2024-12-12

**Authors:** Debora Maria Salimon Pinto, Luciano Garcia Lourenção, Letícia Palota Eid, Maria Amélia Zanom Ponce, Júlio César André, Emilia Batista Mourão Tiol, Bianca Cristina Ciccone Giacon-Arruda, Guilherme de Oliveira Arruda, Maria da Graça Girade Souza, Natália Sperli Geraldes Marin dos Santos Sasaki, Emerson Roberto Santos, William Donegá Martinez, Ana Carolina Santos Costa, Ana Maria Rita Pedroso Vilela Torres de Carvalho Engel, Amilton José da Silva Júnior, Alexandre Lins Werneck, Marise Ramos de Souza, Marlene Andrade Martins, Gabriele Cássia Santos Silva, João Daniel de Souza Menezes, Matheus Querino da Silva, Daniele Alcalá Pompeo

**Affiliations:** 1 Municipal Health Department of São José do Rio Preto, Av. Romeu Strazzi, 199, São José do Rio Preto 15084-010, Brazil; debora.salimon@gmail.com; 2 Ministry of Social Security, Esplanada dos Ministérios, Bloco F, Zona Cívico-Administrativa, Brasília 70059-900, Brazil; lucianolourencao.enf@gmail.com; 3 Institute of Health Sciences, Federal University of Jataí, BR 364, Km 195, 3800, Goiás 75801-615, Brazil; leticiapalota@ufj.edu.br (L.P.E.); marise@ufj.edu.br (M.R.d.S.); marleneandrade@ufj.edu.br (M.A.M.); 4 Postgraduate Nursing Program, Faculty of Medicine of São José do Rio Preto, Av. Brigadeiro Faria Lima, 5416, São José do Rio Preto 15090-000, Brazil; amelinha_famerp@yahoo.com.br (M.A.Z.P.); emilia.tiol@edu.famerp.br (E.B.M.T.); maria.souza@edu.famerp.br (M.d.G.G.S.); natalia.geraldes@famerp.br (N.S.G.M.d.S.S.); alexandre.werneck@famerp.br (A.L.W.); matheusquirino@hotmail.com (M.Q.d.S.); 5 Center for Studies and Development of Health Education, Faculty of Medicine of São José do Rio Preto (CEDES/FAMERP), Av. Brigadeiro Faria Lima, 5416, São José do Rio Preto 15090-000, Brazil; julio.andre@edu.famerp.br (J.C.A.); emerson.santos@edu.famerp.br (E.R.S.); william.martinez@edu.famerp.br (W.D.M.); a.carolinecosta@hotmail.com (A.C.S.C.); anavilelaengel@gmail.com (A.M.R.P.V.T.d.C.E.); joao.menezes@edu.famerp.br (J.D.d.S.M.); 6 Integrated Health Institute, Federal University of Mato Grosso do Sul, Cidade Universitária, s/n, Campo Grande 79070-900, Brazil; bianca.giacon@ufms.br (B.C.C.G.-A.); guilherme.arruda@ufms.br (G.d.O.A.); gabisccor@gmail.com (G.C.S.S.); 7 Institute of Human Sciences, Paulista University, Av. Pres. Juscelino K. de Oliveira, s/n, São José do Rio Preto 15091-450, Brazil; amilton.silva.jr@gmail.com

**Keywords:** mental health, occupational health, job satisfaction, mental health services, COVID-19, health personnel

## Abstract

Background and aims: The COVID-19 pandemic significantly impacted the mental health of healthcare professionals, especially those working in Psychosocial Care Centers (CAPS), which are crucial services in the Brazilian mental health system. This study aimed to investigate the association between job satisfaction, workload, and psychological distress among CAPS professionals during the pandemic. Methods: A cross-sectional study was conducted with 53 professionals from seven CAPS. The Workload Impact Scale (IMPACTO-BR) and Job Satisfaction Scale (SATIS-BR), the General Health Questionnaire (GHQ-12), and a sociodemographic questionnaire were used. Descriptive and analytical statistical analyses were performed. Multiple linear regression analysis was conducted to examine the relationship between job satisfaction, workload, and psychological distress. Results: Professionals reported moderate satisfaction (3.67 ± 0.45) and mild workload (1.82 ± 0.63). One-third of the sample showed scores indicative of psychological distress. Multiple linear regression analysis revealed that workload (*p* = 0.0025) and low job satisfaction (*p* = 0.0495) were significantly associated with psychological distress. Conclusions: Low job satisfaction and high professional workload were predictive variables of psychological distress. These findings highlight the need for investments in promoting the quality of life at work for mental health professionals, especially during crises. The implications for human resource management and public policy development emphasize the importance of an integrated approach that considers the well-being of professionals for the effectiveness and sustainability of the psychosocial care model.

## 1. Introduction

The COVID-19 pandemic profoundly impacted the mental health of healthcare professionals, especially those on the front lines [1,2,3,4,5]. A comprehensive meta-analysis by Wu et al. (2021) [6] demonstrated a global prevalence of 31.4% for anxiety and 33.7% for depression in the general population during the pandemic, which is significantly higher than pre-pandemic rates. For healthcare professionals, the impact has been even more pronounced, with Pappa et al. (2020) [7] reporting prevalence rates of 23.2% for anxiety and 22.8% for depression among healthcare workers during the pandemic. In Psychosocial Care Centers (CAPS), the demand for care increased significantly, exposing these professionals to a high risk of psychological distress [8,9].

Psychological distress, as conceptualized in the Diagnostic and Statistical Manual of Mental Disorders, Fifth Edition (DSM-5) [10], encompasses a constellation of symptoms that may include anxiety, depression, irritability, and concentration difficulties, among others. While not a specific diagnosis, psychological distress serves as a significant indicator of potential mental disorders and is strongly correlated with elevated levels of stress and anxiety. This construct is particularly relevant in assessing the mental health impact of high-stress occupational environments, such as those experienced by healthcare workers during the pandemic. Indeed, Lai et al. (2020) [11] found that frontline healthcare professionals exhibited even higher rates of psychological distress, with 44.6% for anxiety and 50.4% for depression.

A comprehensive review by Kisely et al. (2020) [12] identified several key factors contributing to psychological distress among healthcare workers during infectious disease outbreaks, including the COVID-19 pandemic. Demographic factors such as younger age, female gender, and having dependent children have been associated with higher levels of psychological distress [7]. Occupational factors, including direct contact with COVID-19 patients, inadequate training in infection control measures, and lack of experience in managing infectious diseases, have also been linked to increased distress [11].

CAPS are community mental health services that provide intensive and multidisciplinary care to individuals with severe and persistent mental disorders, playing a crucial role in the Brazilian psychiatric reform and deinstitutionalization. These centers conduct individual and group consultations, home visits, and community activities, aiming at psychosocial rehabilitation and social reintegration of users [13]. In this challenging context, CAPS professionals are particularly vulnerable to the development of burnout, a syndrome resulting from chronic work-related stress. Burnout, characterized by emotional exhaustion, depersonalization, and reduced personal accomplishment, can significantly impact the quality of care provided and the mental health of the professionals themselves. Morgantini et al. (2020) [14] reported a burnout prevalence of 51% in a multinational sample of healthcare professionals during the pandemic, identifying factors such as shortages of personal protective equipment, excessive workload, and concerns about virus transmission as significant contributors to burnout. The inclusion of burnout in this manuscript is essential for understanding the factors contributing to psychological distress among mental health workers during the pandemic, as it represents a critical occupational health outcome that may mediate the relationship between work conditions and overall psychological well-being.

Social support, both within the workplace and from family and friends, has emerged as a significant protective factor against psychological distress. Conversely, social isolation due to quarantine measures or fear of infecting loved ones has been associated with increased distress levels [15]. Organizational factors also play a crucial role. Clear communication from leadership, availability of personal protective equipment (PPE), and access to mental health support services have been identified as protective factors [16]. Conversely, rapidly changing protocols, perceived lack of organizational support, and ethical dilemmas related to resource allocation have been associated with increased distress [17].

The impact of the COVID-19 pandemic in Brazil has been heterogeneous, varying significantly across different regions and states due to factors such as population density, healthcare infrastructure, implemented containment measures, and local socioeconomic characteristics [18]. This regional variability directly influences the intensity and nature of challenges faced by mental health professionals in CAPS [19]. In the Brazilian context, Serpa et al. (2022) [20] observed alarming rates of psychological distress among healthcare professionals, with 55.9% reporting symptoms of anxiety and 46.2% reporting symptoms of depression. These numbers are considerably higher than the rates observed in the general Brazilian population during the same period.

In more severely affected regions, the increased demand for mental health services, combined with limited resources and greater exposure to the virus, may exacerbate psychological distress among professionals [21]. This scenario aligns with the aforementioned concept of psychological distress, where prolonged exposure to intense occupational stressors can manifest in symptoms such as elevated anxiety, depression, and concentration difficulties.

Moreover, regional disparities in pandemic response may result in variations in burnout levels among CAPS professionals [22]. In areas with less institutional support or higher infection rates, professionals may experience elevated levels of emotional exhaustion and depersonalization, key components of burnout. This variability underscores the importance of considering regional context when assessing the pandemic’s impact on healthcare professionals’ mental health.

Individual coping strategies and resilience have also been highlighted as important moderators of psychological distress. Healthcare professionals with higher levels of resilience and effective coping mechanisms, such as problem-focused coping, have reported lower levels of distress [23]. These additional predictors of psychological distress underscore the complex interplay of individual, social, and organizational factors influencing the mental health of healthcare professionals during the pandemic.

In this study, we focus on a large city in the interior of São Paulo state, a region that faced significant challenges during the pandemic [24]. This choice allows us to examine how psychological distress and burnout manifest in an urban context with high demand for mental health services, contributing to a more nuanced understanding of the problem on a national scale. However, research investigating the relationship between working conditions, psychological distress, and professional satisfaction among these workers in the pandemic context is still scarce [25].

To date, only two studies have highlighted the presence of stress, anxiety, and burnout in mental health professionals during the pandemic, one in Asia [26] and another in Europe [27], both emphasizing staff shortages and increased workload as relevant risk factors. Specifically for mental health professionals, Johnson et al. (2021) [28] reported high levels of burnout and psychological distress among psychiatrists and other mental health professionals in the UK during the pandemic, highlighting the unique vulnerability of these professionals who face not only the direct challenges of the pandemic but also an increasing demand for their services. Although there are investigations on the job satisfaction and workload of CAPS professionals [29,30,31,32,33,34], these were not conducted in a critical and unique scenario like the COVID-19 pandemic, highlighting an important gap in the literature.

The literature has consistently pointed to working conditions as fundamental indicators of the quality of care provided [35]. Understanding and improving these conditions is essential not only to enhance clinical outcomes and patient well-being but also to ensure the mental health and satisfaction of professionals. This study, therefore, seeks to provide insights into the formulation of support and intervention strategies that can promote the well-being of mental health professionals in crisis scenarios.

Given this context, the present study aims to analyze satisfaction and workload as predictive factors for psychological distress among CAPS professionals during the COVID-19 pandemic.

## 2. Materials and Methods

### 2.1. Study Design

A quantitative, cross-sectional, and analytical study approach was adopted. The STROBE (Strengthening the Reporting of Observational Studies in Epidemiology) tool [36] was used for organizing and presenting this research report.

### 2.2. Research Setting

This research was conducted in seven CAPS, including two CAPS II for adults, three CAPS for children, one CAPS Alcohol and Drugs II, and one CAPS Alcohol and Drugs III, located in a large city in the interior of São Paulo State, Brazil, with an approximate population of 470,000 inhabitants. CAPS II serve adults in municipalities with populations exceeding 70,000; CAPS AD II and III specialize in alcohol and drug treatment, with type III offering 24 h services; CAPSi cater to children and adolescents.

### 2.3. Population, Selection Criteria, and Sample Definition

The study population consisted of 98 healthcare professionals working in the 7 CAPS, providing direct patient care. Inclusion criteria were established as being an employee with an employment contract with the maintaining entity and working in the service for a minimum period of six months. No exclusion criteria were applied based on a history of psychiatric conditions or personal situations such as intimate partner violence. This study adopted an inclusive approach, focusing on professional and length of service criteria to ensure a comprehensive representation of the CAPS workforce. The presence or absence of psychiatric conditions was not determined as part of the selection process, as this study aimed to capture the diversity of experiences within the professional population. This approach acknowledges the complex interplay between personal history and professional performance in mental health settings. Excluded were workers not performing direct patient care activities (administrative agents, cleaning assistants, kitchen assistants, and security guards) and those on vacation (n = 2) or on sick leave (n = 1). One staff member was excluded from this study due to their termination of employment during the data collection period. This exclusion was necessary to maintain sample consistency, ensuring that all participants were active employees of the CAPS throughout the entire study duration. Another had less than six months of work. This criterion aligns with our research objectives to assess the experiences and perceptions of current CAPS staff members. A total of 12 refused to participate in the research, and 28 did not return the completed or answered instruments.

Given these selection criteria and the operational constraints of conducting research in active healthcare settings during the COVID-19 pandemic, a non-probabilistic convenience sampling method was employed. While this approach limits the generalizability of findings, it was deemed the most appropriate and feasible method to capture the experiences of CAPS professionals under the prevailing circumstances. Thus, the sample consisted of 53 professionals (54.08% of the population), of which 11 (20.75%) were nurses, 9 (16.98%) were doctors, 4 (7.55%) were social workers, 13 (24.53%) were psychologists, 2 (3.77%) were speech therapists, 1 (1.89%) was a physical educator, 2 (3.77%) were occupational therapists, 4 (7.55%) were nursing technicians and assistants, and 7 (13.21%) were workshop instructors.

### 2.4. Instruments Used for Data Collection

To characterize the study participants, an instrument containing personal and professional identification data, closed questions related to stress perception, lifestyle habits, alcohol and tobacco use, psychological or psychiatric follow-up, presence of psychiatric illness, and perception of damages arising from the COVID-19 pandemic, previously validated by five experts in occupational health and/or mental health, was applied.

To assess working conditions, the Satisfaction Assessment Scale for Mental Health Services Team (SATIS-BR) and the Workload Assessment Scale for Mental Health Services Team (IMPACTO-BR) were used. These scales were constructed by the WHO Mental Health Division to specifically evaluate mental health services and were later validated for use in Brazil [37]. These scales were chosen because they have been specifically validated for the Brazilian mental health context, allowing for a more accurate and culturally appropriate assessment of working conditions in CAPS.

The SATIS-BR scale assesses the degree of satisfaction of professionals in mental health services, defined in this study as a positive and gratifying emotional response to the work environment and practices. This satisfaction encompasses the feeling of contentment and happiness at work, in addition to the perception of appreciation and fairness in professional relationships [38]. The abbreviated version of SATIS-BR contains 32 quantitative questions, with responses on a five-point Likert scale, ranging from 1 (very dissatisfied) to 5 (very satisfied). The scale consists of four subscales: (1) satisfaction with the quality of service provided to patients, (2) satisfaction with team participation, (3) satisfaction with working conditions, and (4) satisfaction with relationships with colleagues and superiors. The SATIS-BR demonstrates high reliability, both on the global scale (α = 0.89) and on subscales 1 (α = 0.83), 2 (α = 0.72), 3 (α = 0.77), and 4 (α = 0.63) [37].

The IMPACTO-BR scale assesses the level of perceived workload by mental health professionals in relation to work [37]. Workload was defined as the subjective perception that work demands exceed the resources available to meet them, which can generate stress and other negative effects on the health and well-being of professionals [39]. In its abbreviated version, the scale contains 18 quantitative items, with responses arranged on a five-point Likert scale, ranging from 1 (not at all) to 5 (extremely) [37]. The IMPACTO-BR is composed of three subscales: (1) impact of work on the physical and mental health of the professional, (2) workload in the work environment, and (3) emotional overload and perception of being overwhelmed, demonstrating high internal consistency, both on the global scale (α = 0.87) and on the subscales (α = 0.78, 0.77, and 0.70, respectively) [37].

The SATIS-BR and IMPACTO-BR scales also include open-ended questions (four on satisfaction and three on workload), designed to provide a more comprehensive understanding of workers’ perceptions regarding their satisfaction and workload at work [21]. The open-ended questions included in the SATIS-BR and IMPACTO-BR instruments were as follows: What do you like most about your work? What aspects of your work do you dislike? Do you think the service could be improved? If so, how? What factors do you consider as generating less workload in your job? What factors do you consider as generating greater workload in your job? If you could change something in your work environment, what would you change?

The General Health Questionnaire (GHQ-12) was used to identify symptoms of psychological distress, such as anxiety, depression, concentration difficulties, and sleep disturbances, in the general population, without providing a specific clinical diagnosis [40]. The instrument consists of 12 items that assess the intensity of perceived symptoms in the last few weeks. Responses are provided on a four-point Likert scale. For items indicating mental health problems, options range from 1 = not at all to 4 = much more than usual. For items denoting positive aspects of mental health, the scale ranges from 1 = more than usual to 4 = much less than usual [40]. The GHQ-12 has robust validation in different cultures and populations, including Brazilian samples, showing good reliability and validity indexes in detecting psychological distress [41,42]. In this research, the instrument presented a Cronbach’s alpha of 0.75, indicating adequate internal consistency.

### 2.5. Data Collection

For data collection, the principal researcher held face-to-face meetings with professionals at their respective workplaces during multidisciplinary team meetings. On these occasions, the research objectives were presented, and an invitation for voluntary participation was made. The data collection instruments, along with the printed Informed Consent Form (ICF), were delivered to the managers of each CAPS, who were responsible for making them available to the professionals. In addition, a sealed box was provided for the deposit of completed instruments and the ICF, ensuring the anonymity of the responses.

The data collection was planned so that participants had a period of 15 days to respond to the questionnaires, allowing them to do so at convenient times. After this period, the researcher collected the completed instruments. However, due to low initial adherence, the researcher made new visits to the CAPS in November 2021, January 2022, and March 2022, during team meetings, to reinforce the importance of the research and encourage the participation of professionals. Using this approach, there was an increase in the number of responses, totaling 16 participants, corresponding to 30.1% of the sample.

The total data collection period extended from 1 October 2021 to 30 April 2022. This timeframe coincided with the surge of the Omicron variant of COVID-19, known for its high transmissibility and potential to partially evade immunity [43]. The significance of this timeline lies in the heightened demand for healthcare services and increased operational stress experienced by healthcare professionals during this period. The Omicron surge likely intensified feelings of uncertainty and anxiety among CAPS professionals, influencing this study’s findings on psychological distress and coping mechanisms. Understanding the specific variant context is crucial for interpreting the mental health outcomes observed in this study.

### 2.6. Data Treatment and Analysis and Ethical Aspects

The data were processed and analyzed using StatsDirect software, version 3.3.5. For the sample characterization variables, satisfaction and workload levels, and general health, descriptive analyses were performed, through the calculation of means, medians, and standard deviations. The levels of satisfaction and workload of professionals, both in the global assessment and in the subscales, were measured through the arithmetic mean of the responses. This mean ranges from one to five, with values closer to five indicating higher levels of satisfaction or professional workload [37]. The analysis of the open-ended questions of the satisfaction and workload scales was performed through content analysis, using the thematic categorization technique, which allowed identifying patterns, recurrences, and meanings in the responses, providing an in-depth understanding of participants’ perceptions and experiences. Themes were identified and quantified based on the frequency of their occurrence across all responses. A theme was considered ‘majority’ if it was mentioned by more than 50% of the respondents.

To determine the presence of psychological distress by the GHQ-12, responses were recoded according to the following procedure: responses “1” and “2” were grouped as zero, indicating an absence of psychological distress, while responses “3” and “4” were grouped as one, indicating the presence of psychological distress. The total score, ranging from zero to twelve points, reflects the level of psychological distress, with higher scores indicating greater intensity of distress. A score equal to or greater than three was considered indicative of significant psychological distress, requiring attention [44].

To evaluate the predictive factors for psychological distress, as assessed by the GHQ-12, multiple linear regression analysis was employed. To ensure the adequacy of the regression model, additional checks were performed, including residual analysis, multicollinearity testing, and the Durbin–Watson test [45,46]. The GHQ-12 score served as the dependent variable, while the mean global levels of satisfaction and professional workload were operationalized as independent variables (predictors) in the regression model. Psychological distress was considered a dependent variable, and the mean global levels of satisfaction and professional workload were considered independent variables. A significance level of α = 0.05 was adopted for all statistical analyses. This research was approved by the Research Ethics Committee of the Faculdade de Medicina de São José do Rio Preto on 9 September 2021, under Opinion No. 4.969.390 and Certificate of Presentation and Ethical Appreciation (CAAE) No. 51146821.9.0000.5415.

## 3. Results

The majority of participants were female (64.15%), aged between 31 and 40 years (47.17%), living with a partner (66.04%), and had a specialization as the highest level of education (60.37%).

Most professionals worked in CAPS II 1 (24.53%), in the role of psychologist (24.53%), with professional experience between five and ten years (43.40%) and a workload of 40 h per week or more (56.60%) (Table 1).

Participants reported engaging in weekly physical activities (28.30% one to two times; 30.19% three to four times; 5.66% five to seven times), having leisure (69.81%), not frequently consuming alcoholic beverages (77.36%) or tobacco (94.34%), not undergoing psychological or psychiatric follow-up (62.26%), and not having a diagnosis of psychiatric disorders (69.81%). Regarding the pandemic, 67.92% stated that it affected their mental health in some way.

It is noteworthy that a substantial proportion of our sample (38%) reported ongoing psychological or psychiatric follow-up at the time of this study. This datum stands out as a salient aspect of participant characterization, potentially reflecting a high level of mental health awareness among mental health professionals or indicating the presence of significant psychological challenges within this population.

The overall satisfaction of CAPS professionals was moderate (3.67 ± 0.45). In the subscale analysis, relationships with colleagues received the best evaluation (4.01 ± 0.60), and working conditions received the lowest average (3.42 ± 0.57). The Cronbach’s alpha value for the global scale was 0.89 (Table 2).

In the analysis of open-ended questions, 75% (n = 53) of participants highlighted teamwork as a highly appreciated aspect of the job, mentioning competence, dedication, hospitality, unity, responsibility, and good interpersonal relationships. Other positive points, mentioned by 33 participants (62%), included the high standard of care and the opportunity to work in a free and accessible service that promotes autonomy in patient treatment (mentioned by 18 respondents, 34%). Professionals identified the following factors as generators of professional overload: lack of human resources (mentioned by 40, 75.5%), increased patient demand during the pandemic (38, 71.7%), increased working hours (30, 56.6%), team problems such as lack of involvement and cooperation (25, 47.2%), and excessive bureaucracy (20, 37.7%).

However, 45 professionals (85% of the sample) pointed out dissatisfaction with various aspects of their work environment. Specifically, 30 (56.6%) mentioned inadequate physical infrastructure, 25 (47.2%) pointed out ventilation issues, 28 (52.8%) noted insufficient furniture and space, 35 (66%) highlighted a lack of human and material resources, and 20 (37.7%) reported interpersonal conflicts caused by the lack of proactivity and commitment of some colleagues, as well as indifference and complacency.

The workload of CAPS workers was mild, with a global score of 1.82 ± 0.63. The lowest workload was in physical and mental health, while the highest was in emotional repercussions, including frustration, fatigue, depressive symptoms, and stress (Table 3).

In open-ended questions, professionals pointed out a shortage of human resources, increased patient demand during the pandemic, extended working hours, team issues such as a lack of involvement and cooperation, and excessive bureaucracy as the main factors of professional workload. On the other hand, patient care and teamwork were seen as factors that contributed to a lower workload. To improve the work environment, suggestions included reducing working hours, increasing human resources, greater team receptivity, and more opportunities for discussions.

The analysis of responses to open-ended questions corroborated the quantitative results. For instance, 75% (40/53) of participants mentioned teamwork as a highly appreciated aspect, aligning with the high score on the team relationship subscale of SATIS-BR (4.01 ± 0.60). Similarly, 85% (45/53) expressed dissatisfaction with aspects of the work environment, reflecting the low score on the working conditions subscale (3.42 ± 0.57).

The average GHQ-12 scores were 1.87 ± 2.20, the median was 1.00, and the minimum and maximum values were zero and eight points, respectively. A significant portion of the sample (32.07%) scored three or more points, indicating the presence of psychological distress warranting attention. The highest-scoring questions were the ability to maintain attention to activities and the feeling of being constantly tense and nervous.

Multiple linear regression analysis demonstrated that low job satisfaction and high professional workload were predictive variables of psychological distress, as shown in Table 4.

The beta coefficients (β) presented in Table 4 refer to estimates obtained from multiple linear regression analysis. Each beta coefficient represents the expected change in the dependent variable for each unit increase in the corresponding independent variable, assuming all other independent variables in the model are held constant. A positive beta coefficient indicates a direct relationship between the independent and dependent variable, while a negative coefficient indicates an inverse relationship.

The intercept (y-axis point when x = 0) was 3.859399 with a *p*-value of 0.1285, indicating that no new variables are needed. The correlation coefficients obtained for the global workload means (0.410745) and professional satisfaction (−0.273841), along with the *p*-values, confirm the robustness of the equation: GHQ TOTAL = 3.859399 + 1.393556 (IMPACTO Mean)—1.235461 (SATIS Mean). Individuals with high workload and low job satisfaction are associated with an increase of 1.39 and 1.23 units, respectively, in the measure of psychological distress, assuming all other variables remain constant. The results of this study indicate that the greater the workload and the lower the job satisfaction, the greater the psychological distress of CAPS workers.

## 4. Discussion

The results of this study indicated that CAPS professionals presented moderate job satisfaction levels (3.67 ± 0.45) and low workload (1.82 ± 0.63). These findings are consistent with previous studies conducted in Brazil, which identified similar satisfaction and workload in non-pandemic contexts [29,32,33,34]. However, it is important to highlight that the scenario of this study differs substantially due to the impacts of the COVID-19 pandemic. Therefore, direct comparison with pre-pandemic studies requires caution. Research conducted in Finland highlighted the synergistic effects of job stressors and psychological distress during the pandemic, emphasizing the need to consider these factors in current analyses [47].

One hypothesis for maintaining moderate satisfaction levels during the pandemic may be the support provided by the Municipal Health Department, which implemented a specialized mental health unit for its employees, offering psychiatric, psychological, and social work services. This type of intervention may have helped mitigate the negative effects of the pandemic on professionals’ well-being. Furthermore, the sense of solidarity and strong commitment of workers to continue caring for patients, even under adverse conditions, may have played an important role in maintaining satisfaction [48]. Similar findings were observed in Portuguese workers, where presenteeism and job satisfaction were influenced by organizational support during the pandemic [49].

The relationship with the team was identified as the main factor contributing to job satisfaction, as demonstrated by subscale 4 of SATIS-BR, with the highest mean (4.01 ± 0.60). This result is consistent with previous studies, which highlight the importance of cohesion and support among colleagues in the mental health environment [29,50]. In an environment marked by challenges such as a lack of resources and complexity of care, mutual support among professionals is essential to cope with stress and promote a more satisfactory work environment [48,51,52]. The critical role of team support in reducing psychological distress was also emphasized in studies from Ecuador, underscoring its universal importance in healthcare settings during crises [53].

On the other hand, subscale 3, which assesses working conditions, obtained the lowest mean (3.42 ± 0.57), indicating dissatisfaction with the available physical structure and material resources. The precariousness of working conditions is a recurring issue in community mental health services in Brazil. Although the number of services has grown, financial investments remain insufficient [32,54]. This lack of material and human resources directly affects both the quality of care and the satisfaction of professionals, who often feel limited in their actions and frustrated by not being able to provide adequate care [32].

In CAPS, this reality is even more evident, as professionals develop their know-how on-site, with few evidence-based protocols guiding the execution of actions and procedures. Similarly, for managers, it is not simple to understand the needs of these care facilities, which include large rooms for group activities, provision of snacks and meals for users who remain for long periods, and purchases of materials for crafts, painting, gardening, and construction, instead of syringes, needles, medications, and hospital technologies. Therefore, a closer relationship with managers is necessary [31] so that they can appropriate these health-promoting units, listen to the professionals there, and validate their needs.

Regarding workload, subscale 1 (impact on physical and mental health) presented the lowest mean (1.78 ± 0.75), while subscale 3 (emotional repercussions) had the highest mean (2.12 ± 0.77). These findings indicate that, although professionals are not physically overloaded, they face a strong emotional impact, manifested by frustration, stress, and depressive symptoms. Emotional overload is common in mental health contexts, where professionals are constantly exposed to patients’ suffering and the unpredictability of care situations. In CAPS, professionals need to intensively use their relational and therapeutic skills, adapting to demands and being themselves the tools of work [55]. The increased emotional load during the pandemic, as seen in similar studies, further complicates these challenges [53].

Another aggravating factor was the workload generated by the pandemic, which significantly increased demand in CAPS due to the closure of Primary Health Care units and the redirection of mild cases to mental health services [56]. Additionally, many professionals were relocated to work in vaccination campaigns and other critical care units, generating a feeling of overload and inadequacy, as they could not fully attend to their original responsibilities in CAPS [57].

It is important to note that 38% of participants reported ongoing psychological or psychiatric follow-up at the time of this study. This high prevalence adds a layer of complexity to the interpretation of our results. On one hand, it may indicate heightened mental health awareness among professionals in the field; on the other, it could reflect the cumulative impact of occupational stress on this population.

The results of this study confirmed the relationship between low job satisfaction, high workload, and psychological distress, with 32.07% of professionals presenting psychological distress warranting attention. The literature already points out that inadequate working conditions are predictors of mental health disorders, such as anxiety, depression, and burnout, in healthcare professionals [58,59,60]. During the pandemic, these problems intensified, as demonstrated by a meta-analytic review of 18,000 articles, which identified high rates of insomnia, anxiety, and stress among healthcare workers [58]. This is consistent with findings from studies in Finland and Ecuador, which reported similar mental health challenges among healthcare workers during the pandemic [47,53].

This study advances the existing body of knowledge by examining the relationship between job satisfaction and workload as predictors of psychological distress specifically within the context of Psychosocial Care Centers (CAPS) during the COVID-19 pandemic. While previous research, such as that conducted by Nikunlaakso et al. (2022) [47] and Ruiz-Frutos et al. (2022) [53], highlighted the challenges faced by healthcare professionals in international settings, this study focuses on a unique Brazilian scenario, providing localized insights into how targeted interventions can mitigate the pandemic’s negative impact on workers’ mental health.

These strategies align with recommendations from other studies that emphasize the importance of resilience and coping strategies in managing psychological distress [47]. It is essential for managers to invest in specific training to handle crisis situations effectively, thereby enhancing professionals’ resilience and coping skills. Additionally, promoting regular spaces for case discussion and experience sharing can strengthen mutual support among the team, a factor identified as crucial for job satisfaction.

Finally, reviewing and adjusting the physical and material working conditions should be prioritized, considering the specificities of CAPS. This includes not only improving infrastructure but also ensuring adequate resources for conducting diverse therapeutic activities, essential for the psychosocial care model.

## 5. Limitations

Several limitations of this study should be acknowledged. Firstly, the cross-sectional design and data collection in only one municipality limit the generalizability of the results. Additionally, the use of convenience sampling may introduce selection bias. We acknowledge this sampling approach as a limitation and suggest that future research, when conditions allow, might benefit from employing probabilistic sampling techniques to enhance the representativeness and generalizability of results.

A significant limitation is the lack of explicit differentiation between work-related psychological distress and pre-existing psychiatric conditions. Given that 38% of participants reported ongoing psychological or psychiatric follow-up, this presents a challenge in interpreting our results. We were unable to fully isolate the effects of workload and job satisfaction on psychological distress from those potentially associated with pre-existing conditions.

Furthermore, the absence of scatter plots in the presentation of results limits the direct visualization of the linearity of relationships. However, the additional checks performed support the validity of the linear regression model used.

It is important to acknowledge that the sampling of explanatory (predictor) variables in this study is not exhaustive. Consequently, other factors contributing to psychological distress, beyond job satisfaction and workload, may not have been considered. This limitation aligns with the modern biopsychological model of care, which recognizes the complex interplay of various factors in mental health outcomes. While our results, if maintained after statistical revision, contribute to the existing body of evidence on predictors of psychological distress among frontline healthcare professionals, we cannot establish a comprehensive theory for addressing psychological distress among healthcare workers during the pandemic based solely on these findings. The sampling of factors in our study does not fully encompass the multifaceted nature of psychological well-being as understood in contemporary biopsychological models.

This limitation underscores the need for future research to adopt a more comprehensive approach, incorporating a wider range of potential predictors and considering the complex interactions between biological, psychological, and social factors in the development and management of psychological distress among healthcare professionals.

## 6. Practical Implications

The findings of this study have significant practical implications for human resource management and public policy development, particularly within mental health services during crises such as the COVID-19 pandemic. They highlight the urgent need for interventions to improve working conditions in CAPS, prioritizing the expansion of human and material resources, as well as offering emotional support and continuous training to professionals.

By demonstrating the importance of interventions focused on improving working conditions and providing continuous psychological support, this study establishes a solid foundation for implementing strategies that promote resilience and well-being among mental health professionals as an integral part of organizational culture. Additionally, adopting flexible schedules and role rotation can further help reduce emotional overload, thereby enhancing the overall effectiveness and sustainability of mental health services [49].

Investing in workers’ mental health is essential to ensure the quality of care for users. Although the pandemic has been controlled, its lasting effects on the mental health of CAPS professionals should not be underestimated.

## 7. Directions for Future Research

Future research could benefit from a more nuanced approach, possibly incorporating baseline psychiatric assessments or statistically controlling for ongoing psychiatric follow-up, to better isolate the specific effects of work-related variables on psychological distress.

Multicenter studies are recommended, covering different regions of Brazil, for a broader understanding of the working conditions and mental health of professionals working in community mental health services. These studies can guide the creation of more effective strategies to reduce workload and increase satisfaction, promoting a healthier work environment and, consequently, better quality care for users.

Future studies are indispensable to develop prevention, monitoring, and treatment strategies for mental disorders in this group. The need for broader, multicenter studies is echoed in the literature, which suggests that such research could offer more comprehensive insights into the mental health challenges faced by healthcare workers globally during crises [49].

Additionally, future research could explore the interaction between occupational factors and personal variables, such as psychiatric history, family situation, and socioeconomic status, in determining psychological distress among mental health professionals. Longitudinal studies would be particularly valuable in elucidating the causal relationships between these factors.

## 8. Conclusions

CAPS professionals showed moderate job satisfaction and a low workload. Team relationships contributed to the highest satisfaction, and working conditions contributed to the lowest satisfaction. Among the assessed aspects of workload, emotional repercussions exhibited the highest mean score. This indicates that, although the overall workload was considered low, CAPS professionals experienced a significant emotional impact, primarily manifested through feelings of frustration, stress, and depressive symptoms. This elevated emotional burden stands out as a critical aspect of the occupational demands faced by these professionals. Regarding general health, the results showed that one-third of participants were classified with psychological distress warranting attention. Low job satisfaction and high professional workload were predictive variables of psychological distress.

However, it is crucial to acknowledge that this study does not exhaustively encompass all factors that may contribute to psychological distress. Our sampling of predictor variables, while significant, does not fully capture the multifaceted nature of psychological well-being as understood in contemporary biopsychological models.

These findings underline the need for greater investment in promoting mental health and quality of life at work, addressing the deleterious effects that occurred during epidemic/pandemic outbreaks. Furthermore, they highlight the importance of future research adopting a more comprehensive approach, incorporating a wider range of potential predictors and considering the complex interactions between biological, psychological, and social factors.

This study provides crucial evidence for the development of public mental health policies, especially in the post-pandemic context. The results emphasize the importance of an integrated approach that considers not only the health of service users but also the well-being of the professionals who attend to them. Investing in the mental health and quality of life at work of CAPS professionals is fundamental to ensuring the effectiveness and sustainability of the psychosocial care model in Brazil.

While our findings contribute to the existing body of evidence on predictors of psychological distress among frontline healthcare professionals, we recognize that we cannot establish a comprehensive theory for addressing psychological distress among healthcare workers during the pandemic based solely on these results. Future research should seek a more holistic understanding of these factors to inform more effective policies and interventions.

## Figures and Tables

**Table 1:** Distribution of Psychosocial Care Center professionals included in the sample, according to professional variables (n * = 53). São José do Rio Preto, SP, Brazil, 2022.

Sample Characterization		n *	% ^†^
Work Location	CAPS AD III ^‡^	13	24.53
	CAPS AD II ^§^	4	7.55
	CAPSi 1 ^||^	3	5.66
	CAPSi 2 ^¶^	9	16.98
	CAPSi 3 **	9	16.98
	CAPS II 1 ^††^	14	26.41
	CAPS II 2 ^‡‡^	1	1.89
Role performed	Role Craftsman	7	13.21
	Social Worker	4	7.55
	Physical Educator	1	1.89
	Nurse	11	20.75
	Speech Therapist	2	3.77
	Doctor	9	16.98
	Psychologist	13	24.53
	Nursing Technician	4	7.55
	Occupational Therapist	2	3.77
Work Experience	Less than 5 years	15	28.30
	Between 5 and 10 years	23	43.40
	More than 10 years	14	26.41
	Did not respond	1	1.89
Weekly Workload	Up to 30 h	7	13.21
	Between 30 and 40 h	16	30.19
	40 h or more	30	56.60

* n = sample size; ^†^ % = percentage; ^‡^ CAPS AD III = Psychosocial Care Center Alcohol and Drugs III; ^§^ CAPS AD II = Psychosocial Care Center Alcohol and Drugs II; ^||^ CAPSi 1 = Psychosocial Care Center Child 1; ^¶^ CAPSi 2 = Psychosocial Care Center Child 2; ** CAPSi 3 = Psychosocial Care Center Child 3; ^††^ CAPS II 1 = Psychosocial Care Center Adult 1; ^‡‡^ CAPS II 2 = Psychosocial Care Center Adult 2.

**Table 2:** Results of the satisfaction of professionals with work at the Psychosocial Care Center, using the SATIS-BR * scale (n ^†^ = 53). São José do Rio Preto, SP, Brazil, 2022.

Satisfaction Level	Mean (SD ^‡^)	Minimum	Maximum	Cronbach’s Alpha
Global	3.67 (0.45)	2.66	4.69	0.89
Quality of service provided	3.79 (0.64)	2.20	4.90	0.89
Team/Service Participation	3.64 (0.48)	2.57	4.86	0.62
Working Conditions	3.42 (0.57)	2.20	4.50	0.76
Relationships with Team	4.01 (0.60)	2.00	5.00	0.53

* SATIS-BR = Satisfaction Assessment Scale for Mental Health Services Team—Brazilian Version; ^†^ n = sample size; ^‡^ SD = standard deviation.

**Table 3:** Results of the workload of professionals with work at the Psychosocial Care Center, using the IMPACTO-BR * scale (n ^†^ = 53). São José do Rio Preto, SP, Brazil, 2022.

Workload Level	Mean (SD ^‡^)	Minimum	Maximum	Cronbach’s Alpha
Global	1.82 (0.63)	1.06	3.33	0.92
Impact on physical and mental health	1.78 (0.75)	1.00	3.60	0.85
Impact of work on team	1.82 (0.65)	1.00	3.50	0.76
Emotional repercussions	2.12 (0.77)	1.00	3.80	0.81

* IMPACTO-BR = Workload Assessment Scale for Mental Health Services Team—Brazilian Version; ^†^ n = sample size; ^‡^ SD = standard deviation.

**Table 4:** Results of the multiple linear regression test, considering the satisfaction and workload of professionals with work at the Psychosocial Care Center (n * = 53). São José do Rio Preto, SP, Brazil, 2022.

Variables	β ^§^	r ^||^	*p*-Value ^¶^
Intercept	3.9		0.13
IMPACTO-BR Mean ^†^	1.39	0.41	0.003
SATIS-BR Mean ^‡^	−1.24	−0.27	0.05

* n = sample size; ^†^ IMPACTO-BR = Workload Assessment Scale for Mental Health Services Team—Brazilian Version; ^‡^ SATIS-BR = Satisfaction Assessment Scale for Mental Health Services Team—Brazilian Version; ^§^ β = regression coefficient; ^||^ r = correlation coefficient; ^¶^ *p*-value = multiple linear regression test.

## Data Availability

The datasets generated during and/or analyzed during the current study are available from the corresponding author upon reasonable request.

## Public Involvement Statement

The was no public involvement in any aspect of this research.

## Guidelines and Standards Statement

This manuscript was drafted against the STROBE (Strengthening the Reporting of Observational Studies in Epidemiology) [19].
